# A new species of *Pentamera* Ayres, 1852 from the Brazilian coast (Holothuroidea, Dendrochirotida, Phyllophoridae)

**DOI:** 10.3897/zookeys.634.9769

**Published:** 2016-11-21

**Authors:** Jéssica Prata, Martin L. Christoffersen

**Affiliations:** 1 Universidade Federal da Paraíba, Programa de Pós-Graduação em Ciências Biológicas (Zoologia), Laboratório de Invertebrados Paulo Young (LIPY), Cidade Universitária, S/nº 58059-900, João Pessoa, PB, Brasil; 2 Departamento de Sistemática e Ecologia, Campus I, Universidade Federal da Paraíba, Campus I, CEP 58051-900, João Pessoa, PB, Brasil

**Keywords:** Sea cucumber, southwest Atlantic Ocean, taxonomy

## Abstract

*Pentamera paraibanensis* **sp. n.** is described from more than 3000 specimens as a new species of Phyllophoridae from northeast Brazil, in the tropical southwestern Atlantic. It is distinguished from its congeners by the shape of the calcareous ring with moderate posterior processes, and by the tables in the body wall with a usually quadrilocular disc and a low, toothed spire composed of two pillars. The body is brown, with the tube feet in double rows per ambulacrum, and tentacles more lightly colored. The species was found inside rodoliths in large numbers. This paper contains a morphological description of the specimens, and an account of their habitat characteristics.

## Abstract (pt)

*Pentamera paraibanensis* **sp. n.** é descrita com cerca de 3000 espécimes como uma nova espécie de Phyllophoridae proveniente do Nordeste do Brasil, no Atlântico Sul tropical. Distingue-se das outras espécies do gênero pela forma do anel calcário com processos posteriores moderados, e pelas torres da parede do corpo com um disco geralmente quadrilocular e uma espira curta composta por dois pilares, denteada no ápice. O corpo é marrom, os pés ambulacrais estão dispostos em fileiras duplas por ambúlacro, e tentáculos tem coloração mais clara. A espécie foi encontrada em grande quantidade dentro de rodolitos. Esse trabalho contém uma descrição morfológica dos espécimes, e informações sobre as características de seu habitat.

## Introduction

The order Dendrochirotida contains most of the world’s described holothuroids. In Brazilian waters, it is represented by the families Psolidae, Cucumariidae, Sclerodactylidae and Phyllophoridae (Tommasi 1974, Tommasi et al. 1988a, b, Martins et al. 2012, Ventura et al. 2012). Phyllophoridae (sensu Pawson and Fell 1965) have 10 to 25 tentacles, with tube feet scattered over the entire body or restricted to the radii and a calcareous ring complex with usually radial plates of medium to large size, subdivided into several pieces (Thandar 1990).

Östergren (1907) considered phyllophorids as a subfamily within the Cucumariidae, based on the arrangement of the tentacles. Later, Heding and Panning (1954) revised the family Phyllophoridae, which they subdivided into several subfamilies. Pawson and Fell (1965) regrouped the families of Dendrochirotida based on the shape of calcareous ring (simple or complex) and tentacle number. That review included within the Phyllophoridae the subfamilies Phyllophorinae, Semperiellinae and Thyoninae. Recently Smirnov (2012) considered Thyoninae as differing from other Phyllophoridae on the basis of the number of tentacles and the morphology of the calcareous ring complex. He raised its rank to family level. Several changes in the taxonomy of the Holothuroidea occurred through time, mainly in Dendrochirotida. However, many questions still remain unresolved until now. As more knowledge is produced, answers to some of these open questions will be provided.

Ayres (1852) erected the genus *Pentamera* to accommodate species with oval body, with tube feet in the radii and ossicles of the body wall that are smaller than those present in *Thyonidium* Düben & Koren, 1846. *Pentamera* includes 19 species, most from the Pacific Ocean. In the Western Atlantic only the type species *Pentamera pulcherrima* Ayres, 1852 was hitherto recorded. Species of *Pentamera* are generally of small size with tube feet restricted to the radii and calcareous ring with moderate to long posterior processes and tables from body wall composed of two pillars (Cherbonnier 1951).

Only seven species of Phyllophoridae were known previously from the Southwestern Atlantic: *Pentamera pulcherrima* Ayres, 1852, *Euthyonidiella occidentalis* (Ludwig, 1875), *Neothyonidium parvum* (Ludwig, 1881), *Stolus cognatus* (Lampert, 1885), *Thyone pawsoni* Tommasi, 1972, *Thyone pseudofusus* Deichmann, 1930 and *Thyone montoucheti* Tommasi, 1971. All these species occur along the Brazilian coast.

Numerous specimens associated with rodholiths from the coast of Paraíba, northeast Brazil, represent a new species.

### Abbreviations

UFPB.ECH
Echinodermata Collection of Federal University of Paraiba


UFSITAB
Echinodermata Collection from Federal University of Sergipe


## Material and methods

The examined material is deposited in the Collection of Invertebrates Paulo Young, of the Department of Systematics and Ecology, Federal University of Paraíba (CIPY/DSE–UFPB), and in the Echinodermata Collection, of the Department of Biosciences, Federal University of Sergipe, Brazil. The methods used to study the specimens followed Rowe and Doty (1977), Pawson et al. (2010), and Thandar (1989). Tissue fragments for the extraction of ossicles were immersed in a 3% solution of sodium hypochlorite, washed five times in distilled water, and rinsed with absolute ethanol. Permanent slides were prepared and were studied by light microscope. Other samples were dried and mounted on metal stubs, coated with gold and observed with FEI Quanta 200F scanning electron microscope. Photographs of specimens were taken using a Canon Powershot A2000IS digital camera, and a Leica MZ12.5 stereomicroscope. Measurements were obtained from fixed specimens.

## Systematics

### Order Dendrochirotida Grube, 1840 Family Phyllophoridae Östergren, 1907 Subfamily Thyoninae Panning, 1949

#### Genus Pentamera

Taxon classificationAnimaliaDendrochirotidaPhyllophoridae

Ayres, 1852

##### Diagnosis.

Small to medium sized. Ten tentacles, with two ventral ones smaller. Tube feet only in the radii, elongated, slightly retractile. Calcareous ring with moderate to long posterior processes. Body wall with tables of two pillars or derivatives of these, sometimes accompanied by plates. Tube feet with large endplates and curved supporting tables varying from low to high spire. Tentacles with rods, plates or rosettes (modified after Deichmann 1941, Lambert 1998).

##### Remarks.

The diagnosis has been modified to include the new species and the fact that the type and some other species have plates in the body wall in addition to tables. *Pentamera paraibanensis* sp. n. has posterior processes of medium size and rosettes in the tentacles.

#### Pentamera paraibanensis sp. n.

Taxon classificationAnimaliaDendrochirotidaPhyllophoridae

http://zoobank.org/B14236B1-3248-4A7A-B113-E0CE8EB0B6EF

Figs 1 , 2 , 3

##### Type specimen.

Holotype, UFPB.ECH-2229, João Pessoa, Paraiba State, Brazil, 7°05'01"S; 34°47'56"W, 10 m, associated to rhodoliths, 9 March 2006.

##### Type locality.

João Pessoa, Paraiba State, Brazil, 7°05'01"S; 34°47'56"W, 10 m, associated with rhodoliths, 9 March 2006.

##### Other type material.

Paratype, UFPB.ECH-2230, João Pessoa, Paraiba State, Brazil, 7°05'01"S; 34°47'56"W, 6 March 2006; Paratype, UFPB.ECH-2061, João Pessoa, Paraiba State, Brazil, 7°03'48"S; 34°45'10"W, 15 m, 21 March 2006; Paratype, UFPB.ECH-2048, João Pessoa, Paraiba State, Brazil, 07°05'05"S; 34°44'21"W, 12 m, 24 June 2005; Paratype, UFPB.ECH-2058, João Pessoa, Paraiba State, Brazil, 7°07'00"S; 34°43'54"W, 14 March 2006; Paratype, UFPB.ECH-2089, João Pessoa, Paraiba State, Brazil, 7°03'50"S; 34°47'19"W, 10 m, 21 March 2006.

##### Additional material.

UFPB.ECH-2088, João Pessoa, Paraiba State, Brazil, 6°59'01"S; 34°47'23"W, 10 m, 6 spec., 7 March, 2006; UFPB.ECH-141, João Pessoa, Paraiba State, Brazil, 6°59'01"S; 34°47'23"W, 100 spec, 7 March 2006; UFPB.ECH-148, João Pessoa, Paraiba State, Brazil, 6°59'00"S; 34°46'41"W, 4 spec, 7 March 2006; UFPB.ECH-1684, João Pessoa, Paraiba State, Brazil, 6°59'01"S; 34°45'12"W, 20m, 1 spec., 7 March 2006; UFPB.ECH-145, João Pessoa, Paraiba State, Brazil, 7°01'02"S; 34°47'55"W, 86 spec, 6 March 2006; UFPB.ECH-149, João Pessoa, Paraiba State, Brazil, 7°01'00"S; 34°46'02"W, 2 spec., 6 March 2006; UFPB.ECH-140, João Pessoa, Paraiba State, Brazil, 7°03'50"S; 34°47'19"W, 165 spec., 21 March 2006; UFPB.ECH-143, João Pessoa, Paraiba State, Brazil, 7°03'50"S; 34°47'19"W, 400 spec., 7 March 2006; UFPB.ECH-150, João Pessoa, Paraiba State, Brazil, 7°03'48"S; 34°45'10"W, 31 spec., 21 March 2006; UFPB.ECH-153, João Pessoa, Paraiba State, Brazil, 7°03'49"S; 34°43'12"W, 31 spec., 21 March 2006; UFPB.ECH-204, João Pessoa, Paraiba State, Brazil, 7°04'24,4"S; 34°47'49"W, 6 m, 42 spec., June 2005; UFPB.ECH-858, João Pessoa, Paraiba State, Brazil, 7°7'25,2"S; 34°6'35,0"W, 23 spec.; UFPB.ECH-857, João Pessoa, Paraiba State, Brazil, 7°8'28,836"S; 34°46'34,118"W, João Pessoa, PB, Brazil, 1 spec., 4 October 2007; UFPB.ECH-2087, João Pessoa, Paraiba State, Brazil, 7°03'49"S; 34°47'19"W, 1 spec., 21 March 2006; UFPB.ECH-205, Picãozinho, North Point, João Pessoa, Paraiba State, Brazil, 1 spec., 12 June 2003; UFPB.ECH-2072, João Pessoa, Paraiba State, Brazil, 7°43'09"S; 34°45'00"W, 1 spec.; UFPB.ECH-2068, Coqueirinho Beach, Conde, Paraiba State, Brazil, 1 spec., 3 June 2008; UFPB.ECH-2059, João Pessoa, Paraiba State, Brazil, 07°07'00"S; 34°43'54"W, 1 spec., 11 March 2006; UFPB.ECH-2053, João Pessoa, Paraiba State, Brazil, 07°05'05"S; 34°44'21"W, 12m, 5 spec., 24 June 2005; UFPB.ECH-2057, Reefs in front of the yacht club, Bessa Beach, João Pessoa, Paraiba State, Brazil, 1 spec., 26 February 2006; UFPB.ECH-2049, João Pessoa, Paraiba State, Brazil, 7°05'01"S; 34°47'56"W, 50 spec., 9 March 2006; UFPB.ECH-2052, João Pessoa, Paraiba State, Brazil, 7°05’S, 10 m, 15 spec., 22 February 2006; UFPB.ECH-2037, Cabo Branco Beach, João Pessoa, Paraiba State, Brazil, 1 spec., 17 September 2001; UFPB.ECH-2038, João Pessoa, Paraiba State, Brazil, 7°03'48"S; 34°45’W, 15m, 5 spec., 21 March 2006; UFPB.ECH-2033, João Pessoa, Paraiba State, Brazil, 7°01'02"S; 34°47'55"W, 10m, 13 spec., 6 March 2006; UFPB.ECH-2030, João Pessoa, Paraiba State, Brazil, 7°05'01"S; 34°47'56” W, 93 spec., 9 March 2006; UFPB.ECH-2031, João Pessoa, Paraiba State, Brazil, 7°05'59"S; 34°46'04"W, 10 m, 226 spec., 14 March 2006; UFPB.ECH-1683, João Pessoa, Paraiba State, Brazil, 7°05'05.1"S; 34°44'21"W, 12 m, 14 spec., 24 June 2005.

##### Diagnosis.

Small body, reaching 7 mm, anterior and posterior ends slightly upturned. Color brown in life and in alcohol, tube feet light brown to white. Tube feet only in the radii. Tentacles ten, branched, two ventral ones smaller. Skin thin, smooth. Body wall ossicles comprise oval tables (with disc up to 64 µm long) with four central holes, sometimes more elongated and also with smaller holes marginally and smooth multilocular plates; spire low, with two short pillars ending in 2–3 blunt teeth. Tube feet with supporting plates, curved support tables of variable height, and endplates. Tentacles with rosettes and rods. Introvert with rosettes.

##### Etymology.

The species epithet is derived from the name of the State where it was collected (Paraíba State, Brazil).

##### Description of holotype.

**Figure 1.**
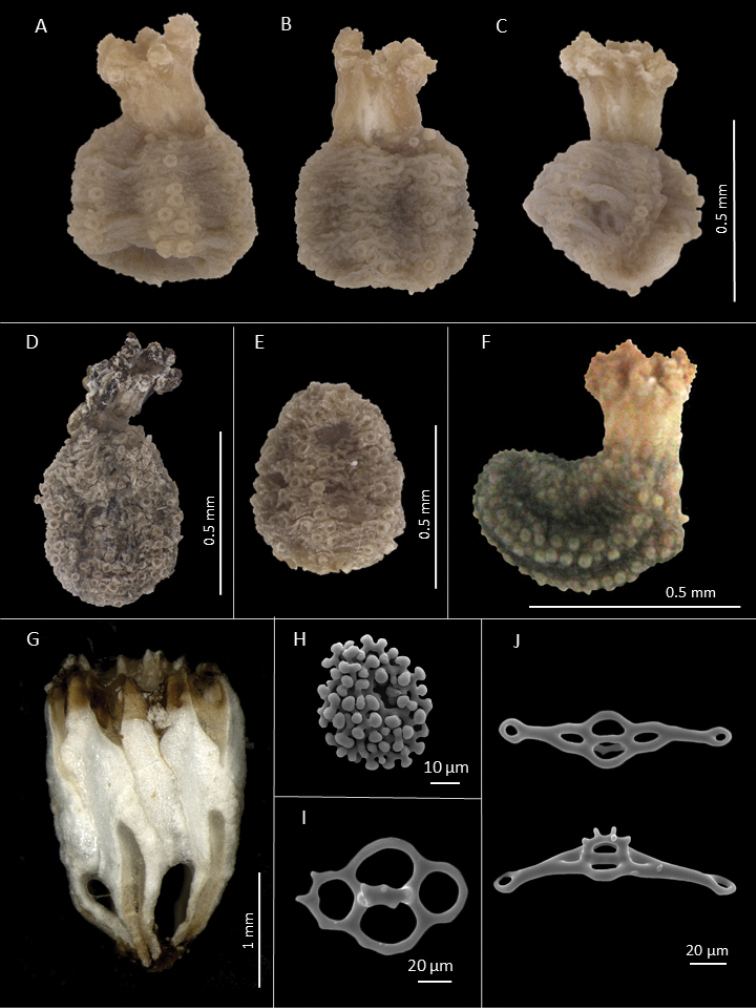
*Pentamera paraibanensis* sp. n. External view of holotype, **A** Dorsal **B** Ventral **C** Lateral **D** External view of specimen UFPB.ECH-2048, **E** External view of specimen UFPB.ECH-2058 **F** External view of specimen UFPB.ECH-2089. **G** Calcareous ring **H** Rosette of tentacles **I** Table from body wall **J** Support tables from tube feet.

Specimen (female) small, globiform, slightly curved, length along the body 7 mm and breadth in mid-body 3 mm (Figure 1A, B, C). Preserved coloration brown, podia and tentacles light brown to white. Mouth upturned; anus terminal with five small papillae and five delicate anal teeth (Figure 3J). Tentacles extended, ten, well-branched, largest about 1.5–2 mm long, two ventral ones smaller. Tube feet restricted to radii, in double rows, longer in the ventral radii, shorter dorsally. Interambulacra usually naked, without papillae, warts or tubercles. Skin smooth, slightly translucid, with numerous small ossicles. Introvert thin, short, without tube feet.

Calcareous ring complex, not fragmented, with posterior processes elongated (Figure 1G). Radial plates longer than interradial, approximately 2 mm high and 0.5 mm wide, anterior part bifid, posterior processes with small pieces; interradial plates triangular anteriorly, with posterior margin convex, 1.3 mm high and 0.3 mm wide. Polian vesicle single, short, saccular, located slightly to left of ventral mesentery; stone canal thin, straight, elongated; madreporite well calcified, bean-shaped. Gonads in one tuft of several tubules attached anteriorly, unbranched but forming several saccules along the tubule, filling the entire body cavity, full of eggs in various stages of development. Longitudinal muscles thin; retractors also thin, more delicate. Respiratory trees confined to posterior quarter of body, with short branches.

**Figure 2.**
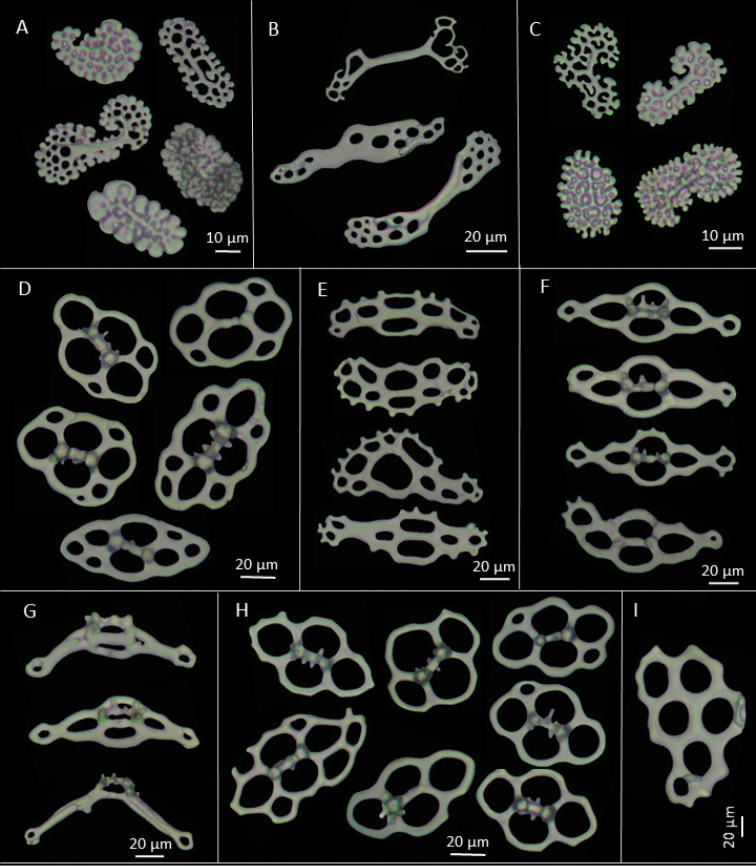
*Pentamera paraibanensis* sp. n. **A** Rosettes from tentacles **B** Rods from tentacles **C** Rosettes from introvert **D** Tables from dorsal body wall **E** Support plates from dorsal tube feet **F** Base of support tables from dorsal tube feet **G** Support tables from dorsal tube feet **H** Tables from ventral body wall, the more elongated table was found near the anus **I** Large plate of tube feet.

**Figure 3.**
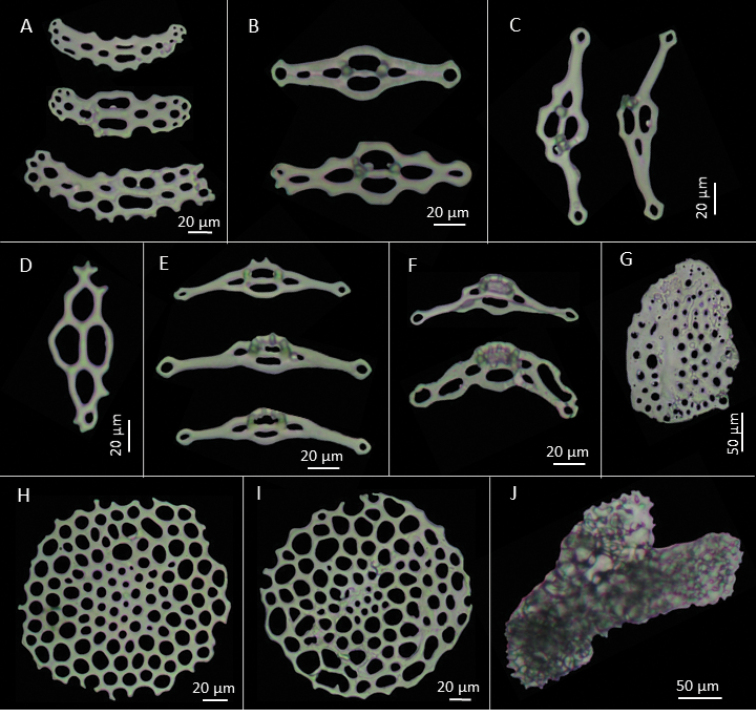
*Pentamera paraibanensis* sp. n. **A** Support plates from ventral body wall **B** Base of support tables from ventral tube feet **C** Curved base of support tables from ventral tube feet **D** Support plate of tube feet **E** Support tables from ventral tube feet **F** Curved support tables from ventral tube feet **G** Large plate from body wall near the anus **H** Endplate from dorsal tube feet **I** Endplate from ventral tube feet **J** Anal tooth.

Characteristic ossicles of body wall as oval tables with disc of usually four perforations and a low spire of two pillars (Figures 1I and 2D and H), 50–70 µm long and 20–30 µm high, ending in 2–3 teeth. Other body wall ossicles include some irregular smooth plates (Figure 3G). Tube feet ossicles of three types, supporting tables with curved disc with four central holes and 1–3 holes at ends, disc 128 µm long and spire 30 µm high (Figures 1J; 2F–G, and 3B–F). Elongate perforated plates, 99 µm long and 40 µm wide (Figure 2E and 3A), and rounded endplates with central perforations smaller than others, about 170 µm in diameter (Figure 3H–I). Some large plates also occur near the podia (Figure 2I). Introvert with rosettes only (Figure 2C). Tentacles with rods of various sizes, some delicate, with perforations at each end, some curved, others with four arms; irregular perforated plates, oblong, straight to slightly curved, medial perforations larger (Figure 2B) and rosettes similar to those of introvert (Figure 1H, 2A).

##### Morphometry.

(See Table 1 below). Rosettes of tentacles are larger than those of introvert. In general, ossicles from dorsal surface larger than those from ventral surface. Dorsally, tables of anterior region are larger than of posterior region but their spire is lower. Endplates are also larger anteriorly. Supporting tables of the podia are smaller in posterior region, but are wider and higher anteriorly. On ventral surface, the tables from anterior part are slightly larger than posteriorly. Endplates of the postero-ventral surface are larger than the anterodorsal surface, about 140 µm in diameter. The supporting tables are slightly larger and taller anteriorly, but wider posteriorly (108.08 × 26.51 × 17.47 µm). The supporting plates from the posterior region are larger, approx. 103 µm.

**Table 1.** Ossicle morphometry of *Pentamera paraibanensis* sp. n. SD, standard deviation; N, number of ossicles measured.

Tentacle						Introvert			
	Rosette Length (µm)	Width (µm)	Rods Length (µm)	Width (µm)		Rosette Length (µm)	Width (µm)		
Mean	44.1	25.84	89.6	8.36		34.18	20.88		
SD	11.2	6.17	38.48	3.83		8.41	4.05		
N	40	40	40	40		40	40		
Anterior region dorsal									
	Table			Endplate	Supporting tables		Supporting plates		
	Length (µm)	Width (µm)	Height (µm)	Diameter (µm)	Length (µm)	Width (µm)	Height (µm)	Length (µm)	Width (µm)
Mean	64.05	43.98	15.76	136.58	108.59	25.92	19.76	109.6	34.3
SD	6.38	4.11	3.28	18.14	9.64	3.39	3.58	14.8	5.65
N	40	40	40	20	30	30	30	20	20
Posterior region dorsal									
	Table			Endplate	Supporting tables			Concave plates	
	Length (µm)	Width (µm)	Height (µm)	Diameter (µm)	Length (µm)	Width (µm)	Height (µm)	Length (µm)	Width (µm)
Mean	60.43	42	16.84	120.86	99.58	30.5	20.09	72.45	55.65
SD	5.58	4.34	2.51	54	8.52	6.41	2.97	9.45	10.45
N	40	40	40	10	30	30	30	15	15
	Supporting plate								
	Length (µm)	Width (µm)							
Mean	83.3	29.4							
SD	11.59	4							
N	10	10							
Anterior region ventral									
	Table			Endplate	Supporting tables		Supporting plates		
	Length (µm)	Width (µm)	Height (µm)	Diameter (µm)	Length (µm)	Width (µm)	Height (µm)	Length (µm)	Width (µm)
Mean	60.78	42.21	15.85	114.33	109.17	25.28	19,88	101,26	26,95
SD	5.73	4.71	2.75	14.96	8,37	3,65	3,13	22,91	4,09
N	40	40	40	10	30	30	30	10	10
Posterior region ventral									
	Table			Endplate	Supporting tables		Supporting plates		
	Length (µm)	Width (µm)	Height (µm)	Diameter (µm)	Length (µm)	Width (µm)	Height (µm)	Length (µm)	Width (µm)
Mean	58.14	40.57	18.37	140.46	108.08	26.51	17.47	103.34	28.06
SD	5.26	4.17	3.69	16.3	11.81	4.69	2.46	10.13	6.95
N	40	40	40	10	35	35	35	25	25

##### Description of paratypes.

The paratypes are from 0.4 to 1 cm long. The ossicles of the body wall and other parts of the body are similar. Some tables are more elongated or have more than four perforations. The color varies from light to dark brown. Some specimens have their body dark brown and their tube feet light brown (Figures 1D–F).

##### Color variations.

A total of 3225 specimens was examined, measuring 3–13 mm long and 3–3.5 mm wide in the mid part, and 1–2.5 mm at the ends were examined. In general, they all present a curved form, but some specimens are elongated or only slightly curved. The body wall is dark to light brown in color, sometimes with dark spots, the tube feet varying from whitish to yellowish, and the tentacles with translucent peduncles and brown to yellow branches. Most specimens present a brown coloration, with some dark brown spots and whitish tube feet.

##### Distribution.

Bessa beach, reefs of Picãozinho, Cabo Branco beach, in Municipality of João Pessoa; Coqueirinho Beach, in municipality of Conde; with coordinates 6°59'01"S; 34°45'12"W and 7°43'09"S; 34°47'56"W, coast of Paraíba State, Brazil. Species found over the continental platform of the State of Paraíba, Brazil, up to 20 m deep.

##### Habitat.

Most specimens were inside rhodoliths, but some samples were associated with *Halimeda* sp., were part of the phytal of *Hypneia* sp., or came from a rocky bottom.

##### Remarks.

The new species seems to shed the calcareous ring when submitted to stress. Some specimens were without the tentacles and calcareous ring, and most of them presented tentacles and the calcareous ring totally extended outside the body. This seems a defense tactic of this animal. The specimens studied agree with the diagnosis of genus *Pentamera* as amended by Lambert (1998). They share the structure of the calcareous ring and the type of body wall ossicles with other species currently classified in the genus *Pentamera. Pentamera paraibanensis* sp. n. with its double row of tube feet, body wall with tables with two pillars, and with the shape of the supporting tables and endplates, has parallels with other species of the genus. *Pentamera paraibanensis* sp. n. has similar tables as those of *Pentamera pediparva* and *Pentamera constricta*, but differs from both in the moderate calcareous ring, height of spire of supporting tables of tube feet, presence of rods and rosettes in tentacles and only rosettes in the introvert. In addition, these species have stiff and rough skin, while *Pentamera paraibanensis* sp. n. has soft and smooth skin. We also compared the new species with other species of the genus *Pentamera*, as well as with other species of Phyllophoridae reported from the South Atlantic directly or through specialized literature (e.g., Cherbonnier (1951), Deichmann (1930, 1938, 1941), Lambert (1998), and Tommasi (1969).

The new species *Pentamera paraibanensis* sp. n. differs of *Pentamera beebei* Deichmann, 1938 and *Pentamera zacae* Deichmann, 1938 by the absence of high pillars of the body wall tables; from *Pentamera chierchiae* (Ludwig, 1887) by the absence of rods in the introvert and tables with spinous disc; from *Pentamera chiloensis* (Ludwig, 1887) by the absence of quadrangular base of tables from the body wall, with pillars ending in several teeth; from *Pentamera calcigera* Stimpson, 1851 it can be distinguished by the absence of a dense layer of plates and by the form of the tables from the body wall; from *Pentamera charlottae* Deichmann, 1938 by the absence of small tables from the body wall; from *Pentamera lissoplaca* (Clark, 1924) by the absence of diamond-shaped tables and diminutive tables in the body wall. *Pentamera paraibanensis* sp. n. differs from *Pentamera trachyplaca* (Clark, 1924) by the absence of thick oval knobbed plates; from *Pentamera pseudocalcigera* Deichmann, 1938 by the absence of star-shaped plates in the body wall; and from *Pentamera rigida* Lambert, 1998 it may be clearly distinguished by absence of large thick tables, knobbed plates in introvert and the shape of the calcareous ring.

*Pentamera paraibanensis* sp. n. distinguishes of the other Phyllophoridae species recorded to South Atlantic, *Euthyonidiella occidentalis* (Ludwig, 1875), *Neothyonidium parvum* (Ludwig, 1881), *Stolus cognatus* (Lampert, 1885), *Thyone pawsoni* Tommasi, 1972 and *Thyone pseudofusus* Deichmann, 1930 by the form of the calcareous ring, arrangement of the tube feet on the body, and set of ossicles from body wall.

### Key to the *Pentamera* species

**Table:** 

1	Small to moderate form, cylindrical, podia in 5 bands, oval to elongated tables with four central holes and a short spire 2-pillared	**2**
–	Small to moderate form, U-shaped to curved body, podia in 5 bands, circular to triangular tables, more of four central holes and short to tall spire 2-pilllared	**6**
2	Supporting tables of tube feet with a short to medium spire	**3**
–	Supporting tables of tube feet with a tall spire	***Pentamera charlottae* Deichmann, 1938**
3	Moderate calcareous ring, supporting tables with medium spire	***Pentamera paraibanensis* sp. n**.
–	Long calcareous ring, supporting tables with low spire	**4**
4	oval tables with four central holes, smooth margin, without knobs and a short spire 2-pillared	***Pentamera citrea* (Semper, 1867)**
–	Oval tables with four central holes, with knobs and a short spire 2-pillared	**5**
5	Small oval buttons with 2 central and up to 8 marginal knobs in body wall, tentacles with round to oblong plates with perforations and knobs	***Pentamera montereyensis* Deichmann, 1938**
–	Body wall with thick oval, knobbed plates with meshwork of bumps covering one side, without buttons, tentacles with oblong reticulate plates	***Pentamera trachyplaca* (Clark, 1924)**
6	Small to moderate form, curved, tapering to blunt ends, posterior processes of calcareous ring moderate to long, body wall without triangular ossicles	**7**
–	Moderate form, tapering in the ends, long posterior processes of calcareous ring, body wall with triangular ossicles	**8**
7	Crowded layer of acorn-like cups with 2-pillared, tapering spire rising from a cup-shaped base	***Pentamera zacae* Deichmann, 1938**
–	Without a Crowded layer of acornlike cups with 2-pillared, tapering spire rising from a cup-shaped base	**9**
8	Large, oval to triangular plates, rarely star-shaped tables, supporting tables with moderate spire, introvert with oval plates with serrate edge and blunt spines on surface	***Pentamera pseudocalcigera* Deichmann, 1938**
–	Circular to triangular or star-shaped tables with a wide central spire, supporting tables with low bumpy spire, introvert with elongated to oval plates with numerous bumps and raised central holes	***Pentamera rigida* Lambert, 1998**
9	circular to oval tables, with smooth margin, with four or more central holes and short to tall spire 2-pilllared	**10**
–	Elongated tables, with wavy margin, with four or more central holes and short to tall spire 2-pilllared	***Pentamera constricta* (Ohshima, 1915)**
10	Introvert with plates and/ or rosettes	**11**
–	Introvert with tables	***Pentamera pediparva* Lambert, 1998**
11	Ossicles of body wall in one layer	**12**
–	Ossicles of body wall in two layers	***Pentamera lissoplaca* (Clark, 1924)**
12	Tentacles with rosettes and plates	**13**
–	Tentacles with plates only	**14**
13	Tentacles with rosettes only	***Pentamera chiloensis* (Ludwig, 1887)**
–	Tentacles with rosettes and plates	***Pentamera pseudopopulifera* Deichmann, 1938**
14	Tentacles with irregular to oval perforated plates	**15**
–	Tentacles with elongated diamond-shaped plates with two large central holes, some with bumps or low pillar arch	***Pentamera populifera* (Stimpson, 1864)**
15	Tentacles with irregular perforated plates	***Pentamera pulcherrima* Ayres, 1852**
–	Tentacles with oval perforated plates with a meshwork at center	***Pentamera calcigera* Stimpson, 1851**

## Supplementary Material

XML Treatment for Pentamera

XML Treatment for Pentamera paraibanensis
